# COVID-19 mRNA vaccine effectiveness (second and first booster dose) against hospitalisation and death during Omicron BA.5 circulation: cohort study based on electronic health records, Portugal, May to July 2022

**DOI:** 10.2807/1560-7917.ES.2022.27.37.2200697

**Published:** 2022-09-15

**Authors:** Irina Kislaya, Ausenda Machado, Sarah Magalhães, Ana Paula Rodrigues, Rafael Franco, Pedro Pinto Leite, Carlos Matias Dias, Baltazar Nunes

**Affiliations:** 1 Departamento de Epidemiologia, Instituto Nacional de Saúde Doutor Ricardo Jorge, Lisbon, Portugal; 2 Centro de Investigação em Saúde Pública, Escola Nacional de Saúde Pública, Universidade NOVA de Lisboa, Lisbon, Portugal; 3 Serviços Partilhados do Ministério da Saúde, Lisbon, Portugal; 4 Direção de Serviços de Informação e Análise, Direção-Geral da Saúde, Lisbon, Portugal

**Keywords:** SARS-CoV-2, COVID-19-related hospitalization, Vaccine Effectiveness, COVID-19-related death, Omicron BA.5

## Abstract

We measured vaccine effectiveness (VE) against COVID-19-related severe outcomes in elderly people in Portugal between May and July 2022. In ≥ 80 year-olds, the second booster dose VE was 81% (95% CI: 75–85) and 82% (95% CI: 77–85), respectively, against COVID-19-related hospitalisation and death. The first booster dose VE was 63% (95% CI: 55–70) in ≥ 80 year-olds and 74% (95% CI: 66–80) in 60–79 year-olds against hospitalisation, and 63% (95% CI: 57–69) and 65% (95% CI: 54–74) against death.

Since 15 May 2022, a second booster dose of the coronavirus disease (COVID-19) mRNA vaccine has been rolled out in Portugal for those 80 years and older or residents in long-term care facilities [1], according to the European Centre for Disease Prevention and Control (ECDC) and the European Medicines Agency (EMA) recommendations issued in April 2022 [2]. In July 2022, following the spread of the severe acute respiratory syndrome coronavirus 2 (SARS-CoV-2) Omicron (Phylogenetic Assignment of Named Global Outbreak Lineages (Pangolin) designation B.1.1.529) BA.5 variant across Europe, ECDC and EMA extended the recommendation of the second booster dose to the population aged 60–79 years [3]. However, at the time of this study, this extension has not yet been implemented in Portugal.

We aimed to estimate vaccine effectiveness (VE) of the second booster dose against COVID-19-related hospitalisation and death in the population in Portugal 80 years and older during Omicron BA.5 circulation, from 15 May to 31 July 2022. As a secondary objective, we present VE of the primary series and the first booster dose against COVID-19-related severe outcomes in those aged 60 years and older.

## Study design

We carried out a study based on linkage of electronic health records using the National Health Service User (NHSU) unique numeric identifier to link the databases [4]. The study period covered the period of circulation of Omicron BA.5 [5], from 15 May 2022, when the second booster began to be administrated, to 31 July 2022. The target population included residents in Portugal mainland, aged 60 years and older and eligible for COVID-19 vaccination.

Exclusion criteria were: age registered as more than 110 years, documented SARS-CoV-2 infection within 90 days before the start of the follow-up, being vaccinated with vaccines other than those recommended in Portugal, having an interval between doses other than recommended and not having any contact with the national healthcare service in the past 3 years. We estimated the VE separately for two cohorts: age 60–79 years and ≥ 80 years.

## Outcomes definitions

COVID-19-related hospitalisation was defined as admission to a hospital for at least 24 h, following laboratory-confirmed infection with SARS-CoV-2 and having COVID-19 as the primary diagnosis at discharge (ICD10 coding U071).

COVID-19-related death was defined as death for which COVID-19 was recorded as the cause of death (U071) or deaths that occurred within 30 days after the laboratory-confirmed SARS-CoV-2 infection. We included all death events provided that the laboratory confirmation date occurred during the study period, i.e. even if the death occurred after the study period.

## Exposures definitions

Data on exposure status were obtained from the population-based electronic vaccination registry (VACINAS). Participants without any registered COVID-19 vaccine dose were classified as *unvaccinated*. Participants were classified as vaccinated with the primary series 14 days following vaccine uptake, according to the product characteristics (single dose of Janssen vaccine or two doses of an mRNA vaccine or Vaxzevria). Only mRNA vaccines were used for the booster doses. Participants were classified as *vaccinated with the first booster* 14 days following the uptake of the mRNA vaccine booster dose. Participants were classified as *vaccinated with the second booster dose* 14 days after the second booster mRNA COVID-19 vaccine dose uptake. Those with documented uptake of any dose of COVID-19 vaccine but who did not fit the above definitions were not included in the study.

For the age group ≥ 80 years, we considered second booster dose uptake as the main exposure and the unvaccinated as the reference group. We also estimated relative vaccine effectiveness (rVE), to quantify the additional protection conferred by the second booster relative to other vaccination regimens.

For the cohorts 60–79 years and ≥ 80 years, we report the VE for the first booster dose and for the primary series during the study period, and rVE to compare protection between first booster dose and primary series.

## Statistical analysis

Descriptive statistics were used to characterise participants at baseline by exposure level and at the end of the follow-up period. For each outcome and cohort, VE was estimated as VE = 100% × (1 − HR), where HR stands for confounder-adjusted hazard ratio obtained through a time-dependent Cox regression model, adjusted for age group (5-year bands), sex, municipality-level European deprivation index quintile [6], number of chronic diseases, number of SARS-CoV-2 laboratory tests during the period 2020 to 2022, previous influenza vaccine or pneumococcal vaccine uptake. Complete case analysis was used.

Data analysis was performed with R software, version 4.0.5 (R Foundation, Vienna, Austria). The statistical significance level was set at 5%.

## Description of the participants and events

Among 642,067 enrolled Portuguese residents aged ≥ 80 years, 53.3% received the second booster of a COVID-19 vaccine (Table 1). The coverage with the first booster dose in the age cohort 60–79 years (n = 1,984,107) was 81.4% (Table2).

**Table 1:** Participants' characteristics at baseline by exposure status at the end of the follow-up period, cohort age ≥ 80 years, COVID-19 vaccine effectiveness study, Portugal, 15 May–31 July 2022 (n = 642,067)

	Unvaccinated (n = 51,411)		Primary series (n = 44,284)		First booster (n = 204,362)		Second booster (n = 342,010)	
	n	%	n	%	n	%	n	%
Age in years, median (IQR)	86.0 (82.0–90.0)		85.0 (82.0–88.0)		84.0 (82.0–88.0)		84.0 (82.0–88.0)	
Age group (years)								
80–84	21,320	41.5	20,930	47.3	106,473	52.1	175,554	51.3
85–89	16,285	31.7	14,838	33.5	65,368	32.0	113,156	33.1
90–94	9,410	18.3	6,614	14.9	25,967	12.7	43,222	12.6
≥ 95	4,396	8.6	1,902	4.3	6,554	3.2	10,078	2.9
Male sex	18,065	35.1	14,080	31.8	74,941	36.7	138,906	40.6
Region								
ARS Alentejo	2,315	4.5	2,565	5.8	12,558	6.1	19,618	5.7
ARS Algarve	3,941	7.7	3,190	7.2	9,922	4.9	13,141	3.8
ARS Centro	9,275	18.0	8,455	19.1	41,654	20.4	68,354	20.0
ARS LVT	18,036	35.1	16,513	37.3	74,848	36.6	120,956	35.4
ARS Norte	11,785	22.9	13,054	29.5	64,190	31.4	118,863	34.8
Missing	6,059	11.8	507	1.1	1,190	0.6	1,078	0.3
European deprivation index quintile								
Q1 (least deprived)	6,462	12.6	5,975	13.5	32,086	15.7	56,305	16.5
Q2	6,524	12.7	6,266	14.1	31,689	15.5	51,919	15.2
Q3	6,336	12.3	6,192	14.0	29,000	14.2	50,771	14.8
Q4	12,445	24.2	12,226	27.6	57,324	28.1	97,586	28.5
Q5 (most deprived)	13,585	26.4	13,118	29.6	53,073	26.0	84,351	24.7
Missing	6,059	11.8	507	1.1	1,190	0.6	1,078	0.3
Number of chronic diseases								
0	20,039	39.0	7,012	15.8	26,410	12.9	39,716	11.6
1	10,846	21.1	9,491	21.4	40,555	19.8	66,821	19.5
2	9,411	18.3	10,897	24.6	51,631	25.3	87,130	25.5
3	6,168	12.0	8,395	19.0	42,510	20.8	73,868	21.6
4	3,091	6.0	5,048	11.4	25,607	12.5	44,013	12.9
≥ 5	1,856	3.6	3,441	7.8	17,649	8.6	30,462	8.9
Number of SARS-CoV-2 tests								
0	36,601	71.2	17,492	39.5	75,411	36.9	148,877	43.5
1	6,657	12.9	8,834	19.9	43,415	21.2	69,375	20.3
2	3,068	6.0	5,222	11.8	26,217	12.8	42,123	12.3
3	1,708	3.3	3,224	7.3	16,536	8.1	25,322	7.4
4–9	2,802	5.5	7,399	16.7	34,249	16.8	47,183	13.8
≥ 10	575	1.1	2,113	4.8	8,534	4.2	9,130	2.7
Any other vaccine uptake^a^	8,610	16.7	23,952	54.1	168,146	82.3	311,662	91.1

ARS: regional health administration; COVID-19: coronavirus disease; LVT: Lisbon and Tagus Valley; SARS-CoV-2: severe acute respiratory syndrome coronavirus 2.

^a^ Received at least one of the following vaccines since 2017, influenza vaccine, pneumococcal conjugate vaccines (PCV13, PCV7 or PCV10) and pneumococcal polysaccharide vaccine (PPSV23).

**Table 2:** Participants' characteristics at baseline by exposure status at the end of the follow-up period, age cohort 60–79 years, COVID-19 vaccine effectiveness study, Portugal, 15 May–31 July 2022 (n = 1,984,107)

	Unvaccinated (n = 147,512)			Primary series (n = 220,699)			First booster (n = 1,615,896)		
	n	%		n	%		n	%	
Age in years, median (IQR)	68.0 (63.0–73.0)			67.0 (63.0–72.0)			69.0 (65.0 – 74.0)		
Age group									
60–64	49,832		33.8	78,648		35.6	402,090		24.9
65–69	37,828		25.6	58,750		26.6	451,544		27.9
70–74	33,054		22.4	47,360		21.5	438,185		27.1
75–79	26,798		18.2	35,941		16.3	324,077		20.1
Male sex	69,802		47.3	98,113		44.5	733,273		45.4
Region									
ARS Alentejo	5,626		3.8	8,917		4.0	78,900		4.9
ARS Algarve	15,324		10.4	13,240		6.0	75,145		4.7
ARS Centro	22,902		15.5	35,640		16.1	288,851		17.9
ARS LVT	52,152		35.4	76,948		34.9	556,184		34.4
ARS Norte	32,406		22.0	83,339		37.8	608,265		37.6
Missing	19,102		12.9	2,615		1.2	8,551		0.5
European deprivation index quintile									
Q1 (least deprived)	16,777		11.4	28,032		12.7	238,864		14.8
Q2	17,048		11.6	30,038		13.6	241,030		14.9
Q3	16,564		11.2	31,766		14.4	238,676		14.8
Q4	34,792		23.6	62,347		28.2	464,215		28.7
Q5 (most deprived)	43,229		29.3	65,901		29.9	424,560		26.3
Missing	19,102		12.9	2,615		1.2	8,551		0.5
Number of chronic diseases									
0	81,499		55.2	65,604		29.7	421,596		26.1
1	31,436		21.3	55,312		25.1	420,812		26.0
2	19,093		12.9	47,571		21.6	378,138		23.4
3	9,613		6.5	30,688		13.9	239,821		14.8
4	4,034		2.7	14,192		6.4	105,536		6.5
≥ 5	1,837		1.2	7,332		3.3	49,993		3.1
Number of SARS-CoV-2 tests									
0	86,350		58.5	49,951		22.6	601,190		37.2
1	26,439		17.9	50,811		23.0	360,039		22.3
2	13,366		9.1	36,574		16.6	233,889		14.5
3	7,244		4.9	24,951		11.3	144,098		8.9
4–9	11,813		8.0	49,497		22.4	244,315		15.1
≥ 10	2,300		1.6	8,915		4.0	32,365		2.0
Any other vaccine uptake^a^	15,161		10.3	103,616		46.9	1,143,377		70.8

ARS: regional health administration; COVID-19: coronavirus disease; LVT: Lisbon and Tagus Valley; SARS-CoV-2: severe acute respiratory syndrome coronavirus 2.

^a^ Received at least one of the following vaccines since 2016, influenza vaccine, pneumococcal conjugate vaccines (PCV13, PCV7, or PCV10) and pneumococcal polysaccharide vaccine (PPSV23).

Between 15 May 2022 and 31 July 2022, 1,343 COVID-19-related hospitalisations occurred in the in ≥ 80 year-olds and 740 in the 60–79 year-olds, while the number of deaths was 1,975 and 710, respectively.

### Vaccine effectiveness against COVID-19-related hospitalisation

In the age cohort ≥ 80 years, the VE against COVID-19-related hospitalisation was 56% (95% CI: 46–65) for the primary vaccine series, 63% (95% CI: 55–70) for the first mRNA booster dose and 81% (95% CI: 75–85) for the second mRNA booster dose (Table 3).

**Table 3:** COVID-19-related hospitalisation and death rates by exposure status, and absolute and relative COVID-19 vaccine effectiveness estimates, Portugal, 15 May–31 July 2022 (n = 2,626,174)

Exposure status	Person-years	Events	Rate per 100,000 person-years	VE (95% CI)	rVE second booster and first relative to other vaccination regimens (95% CI)	rVE second booster relative to first booster (95% CI)
**≥ 80 year-olds**						
**Hospitalisation**						
Unvaccinated	10,901	164	1,505	Reference	NA	NA
Primary series	10,491	188	1,792	56 (46–65)	Reference	NA
First booster	61,637	858	1,392	63 (55–70)	16 (1–28)	Reference
Second booster	39,633	133	336	81 (75–85)	53 (43–65)	47 (35–57)
**Death**						
Unvaccinated	10,911	231	2,117	Reference	NA	NA
Primary series	10,503	274	2,609	58 (49–65)	Reference	NA
First booster	61,702	1,209	1,959	64 (57–69)	13 (1–24)	Reference
Second booster	39,646	261	658	82 (77–85)	56 (47–63)	49 (41–56)
**60–79 year- olds**						
**Hospitalisation**						
Unvaccinated	31,482	94	299	Reference	NA	NA
Primary series	48,566	113	233	67 (56–75)	Reference	
First booster	341,824	533	156	74 (66–80)	21 (2–36)	
**Death**						
Unvaccinated	31,491	67	213	Reference	NA	NA
Primary series	48,578	120	247	59 (44–70)	Reference	
First booster	341,875	523	153	65 (54–74)	14 (−5 to 30)	

COVID-19: coronavirus disease; NA: not applicable; rVE: relative vaccine effectiveness; VE: vaccine effectiveness.

The second booster dose was more effective in preventing COVID-19-related hospitalisations when compared either with the primary series (rVE = 53%; 95% CI: 43–65) or the first booster dose (rVE = 47%; 95% CI: 35–57).

In the age cohort 60–79 years, VE for primary series and the first mRNA booster dose against COVID-19-related hospitalisation was, respectively, 67% (95% CI: 56–75) and 74% (95% CI: 66–80), which represents a rVE of 21% (95% CI: 2–36).

### Vaccine effectiveness against COVID-19-related death

For the age cohort ≥ 80 years, we estimated a VE against COVID-19-related death of 58% (95% CI: 50–65) for complete primary vaccination, 64% (95% CI: 57–69) for the first booster dose and 82% (95% CI: 77–85) for the second booster dose.

Uptake of the second mRNA booster dose was associated with a 49% (95% CI: 41–56) reduction in the risk of COVID-19-related death compared with the first booster dose. The protective effects of the first mRNA booster relative to the primary vaccination on preventing COVID-19-related deaths were much lower (rVE = 13%; 95% CI: 1–24).

In the age cohort 60–79 years, we observed a VE of 59% (95% CI: 44–70) and 65% (95% CI: 54–74) for the primary series and the first mRNA booster dose against COVID-19-related death, representing a risk reduction of 14% for COVID-19-related death when comparing first booster dose with the primary vaccine series.

## Discussion

Using a cohort of the population 80 years and older, based on electronic health records linkage, we estimated a high VE of the second mRNA booster dose in preventing COVID-19-related hospitalisations (VE = 81%) and deaths (VE = 82%) during the period of BA.5 circulation in Portugal. Our estimates are comparable to the ones reported for ≥ 50 year-olds during the period of Omicron BA.2/BA.2.12.1 predominance in the United States (VE = 80%; 95% CI: 71–85) [7] and residents of long-term care facilities in Canada (VE = 86%; 95% CI: 81–90) [8].

We observed moderate vaccine-induced protection for both primary vaccination and first booster dose in 60–79 year-olds and in those 80 years and older, ranging between 56% and 74% against COVID-19-related hospitalisation and between 58% and 65% against COVID-19-related mortality. Meaning that individuals with primary vaccination or first booster dose maintain an increased protection against COVID-19 severe outcomes when compared with unvaccinated individuals.

A significant increase in protection against COVID-19-related hospitalisation and mortality compared with the first booster (rVE = 47% for hospitalisation and rVE = 49% for mortality) are in line with rVE of 42% reported for all-cause mortality in Sweden in the period January to March 2022 for those aged 80 years and older [9]. However, our rVE of the second booster against hospitalisations was lower than reported in Israel for people 60 years and older (74%; 95% CI: 48–91) [10]. Nevertheless, comparisons should be made with caution given the differences in eligible age groups, outcomes and epidemiological context in the countries, mainly the fact that the study period for both these studies was January-February 2022, during early stages of Omicron predominance and our study covers the Omicron BA.5 predominance period in Portugal.

Among the limitations of this study, we should mention the possibility of outcome misclassification bias related to the coding of hospitalisations at discharge. If the vaccination status is associated with shorter length of hospitalisation, the hospitalisations could be more up to date at the time of data extraction in the second booster dose group, and this would bias the VE in the null direction, so we would be underestimating the second booster dose VE. Secondly, there is a possibility of residual confounding in VE estimates, as the number of confounders available in electronic health records is limited, and data on mask use and physical distancing are not available at individual level. Thirdly, although in the establishment of the cohorts we included several steps to reduce the number of duplicates or non-real individuals, we cannot rule out the possibility of non-real individuals in the cohorts. Fourthly, we did not perform genetic characterisation of the viruses. Surveillance data indicated that prevalence of the BA.5 lineage in Portugal during the study period ranged between 64 and 97% [5]. Nevertheless, we cannot assume that all infections were caused by the BA.5 lineage. Thus, we cannot completely generalise our results as VE measurements against the BA.5 Omicron variant. Finally, the follow-up time after the second booster dose was limited, so other studies are needed to address waning of vaccine protection.

## Conclusion

For the population aged 80 years and older, our study supports a high VE of the second mRNA booster dose in preventing severe COVID-19-related outcomes, and a relevant increase in protection compared with previous vaccination during Omicron BA.5 predominance. In the group aged 60–79 years, and given that the VE estimates against hospitalisation were lower for the first booster dose compared with early stages of the first booster dose roll-out (74% vs 95%) [11], the recommendation of extending the second booster dose to this age group could have a relevant benefit in terms of COVID-19 impact mitigation.
